# Longevity of Medical Men

**Published:** 1845-05

**Authors:** 


### THE WESTERN JOURNAL.

New Series.—Vol. III.—No. V.

LOUISVILLE, MAY 1, 1845.

### LONGEVITY OF MEDICAL MEN.

The following note was sent to us by Dr. Boling to accompany his essay on the "Health of the Clergy," but reached us too late for our last number. Its humor will amuse, and its facts are worth preserving. We insert it in this place, and are sure that our readers will thank us for the opportunity of enjoying a laugh. Y.

"As an appendix to the catalogue of female writers of eminence who lived to an advanced age, I give below a short list of distinguished members of the medical profession, arranged in the order in which the names occurred to my mind. They are the only ones for whose ages I have examined. They were all highly distinguished, and although the opinions and doctrines advocated by some of them, may have been incorrect, and may have tended but little to the ultimate advancement of the science, still the genius of the authors gave to their opinions for a time a wide spread popularity. It may be maintained that it was not so much to superior abilities, as to the lengthened term of their lives that we are indebted for their labors. But to this my answer is that, in many instances their principal works, the foundation upon which the reputations of the authors were built, were written before the attainment of old age—in some instances, in the earlier years of manhood.

| | Years. | | Years. |
|---|---|---|---|
| Hippocrates, from | 85 to 109 | Haller, | 69 |
| Galen, | 70 | Harvey, | 80 |
| Boerhaave, | 78 | Sir C. Bell, | 67 |
| Cullen, | 78 | Sir A. Cooper, | 73 |
| Sydenham, | 65 | Abernethy, | 66 |
| Brown, (intemperate) | 53 | Malpighi, | 67 |
| Rush, | 68 | Ruysch, | 93 |
| Physick, | 70 | Morgagni, | 89 |
| Broussais, | 66 | Larrey, | 75 |
| Hunter, (John) | 65 | Darwin, | 71 |

"Shall I add to this list the names of Cervantes and Hahnemann? In regard to the former there is perhaps no good reason for classing him with medical authors, unless we are agreed to take the authority of the great Sydenham as conclusive on this point, who, as is known, when consulted by Sir Richard Blackmore as to what books he should study to prepare him for the medical profession, advised him to "read Don Quixote." The formula for the "precious balsam," prepared by the Knight of the rueful countenance, which produced such wonderful effects on his Squire, gives him further claims to a rank in our profession. This is the only thing in his immortal work that I can at this moment call to mind which can be considered as in any way pertaining to practical medicine. Perhaps it would be more correct to class both him and Hahnemann with the satirists. As Don Quixote was written in ridicule of the extraordinary and absurd ideas prevalent in regard to Chivalry, so in all probability was it the original intention of the sly old German, that his "Organon" should be received as a burlesque upon the different medical doctrines and theories which from time to time had replaced one another. "Many wild and ridiculous theories of medicine," he probably said to himself, "have prevailed, and have been the cause of much sarcasm upon the profession. My satire upon these false systems shall be the severest of all. I will do for medicine what Cervantes has done for chivalry"—and lo, the "Organon" appeared! Had it been received, as must have been the original object of the author, the pungent wit expended upon the profession by Le Sage, Smollet, Moliere, and others, sparkling though it was, would have been completely dimmed. The jest of the author was taken as earnest, however, and though his wit was wasted, his pockets were filled. This was probably some consolation for his disappointment, and may have rendered the laugh in his sleeve more merry, till in the years of second childhood, he himself began to believe—received, in other words, that degree of enlightenment which had long since been attained by his proselytes. If the first object for which the work was intended was defeated, it probably advanced as well the authors interest, and served to develope a degree of gullibility in mankind previously unsuspected."

